# Colorectal carcinoma: some reflections on bile flow through the terminal ileum.

**DOI:** 10.1038/bjc.1997.351

**Published:** 1997

**Authors:** M. Stoneham


British Journal of Cancer (1997) 76(1), 136–137

© 1997 Cancer Research Campaign

Letters to the Editor

### Colorectal carcinoma: some reflections on bile flow through the terminal ileum

### Sir

I have just read with great interest the paper by Boutron et al (1996). They suggest that the type of dairy product might be the important factor with regard to prevention of colorectal tumours.

It is widely believed that bile salts may have some role in colorectal carcinoma (CRC) (Nagengast, 1988; Garewal et al, 1996). The enzyme diamine oxidase (DAO), which is a major catabolic enzyme for histamine, is found in highest concentration

**Figure 1** Summary of possible DAO effect in the terminal ileum in the ileal mucosa (Lessof et al, 1990). DAO is thought to be involved in the possible inhibition of colonic mucosal proliferation (Kusche et al, 1988). It has been shown that DAO activity can be reduced in vitro by several factors including detergent (Sattler et al, 1987), and bile salt (Stoneham et al, 1993). Alcohol has also been thought to inhibit DAO in vivo (Wantke et al, 1993). There appears to be a limit to cholesterol solubility in the bile salt micelle (Chijiwa and Nagai, 1987*a*,*b*) that might lead to variable amounts of free bile salt in the lumen of the terminal ileum with different dietary fat intakes. These statements are summarized in Figure 1.

Could these mechanisms in vivo go some way to explain the geographical variations in colorectal cancer incidence that have been correlated with different regional dietary patterns (Armstrong and Doll, 1975; Bingham, 1990), and also the possible link with cholecystectomy? (Ekbom et al, 1993; Giovanni et al, 1993). Could the possible inhibition of DAO by alcohol help in understanding the role of alcohol ingestion in colorectal carcinoma (Hirayama, 1989)? If the importance of DAO inhibition is established in vivo, could the susceptibility of certain families to colorectal cancer (Petersen, 1995; Winawer et al, 1996) be partly related to genetic variants of DAO?

*Michael Stoneham*
*UHCE*
*Institute of Health Sciences*
*Old Road*
*Oxford OX3 7LF*

### REFERENCES

Armstrong B and Doll R (1975) Environmental factors and cancer incidence and mortality in different countries, with special reference to dietary practices. *Int J Cancer* **15**: 617–631

Bingham SA (1990) Diet and large bowel cancer. *J Roy Soc Med* **83**: 420–421

Boutron MC, Faivre J, Marteau P, Couillart C, Senesse P and Quipourt V (1996) Calcium, phosphorus, vitamin D, dairy products and colorectal carcinogenesis: a French case–control study. *Br J of Cancer* **74**: 145–151

Chijiwa K and Nagai M (1987*a*) Interaction of bile salt monomer and cholesterol in the aqueous phase. *Biochim Biophys Acta* **1001**: 111–114

Chijiwa K and Nagai M (1987*b*) Bile salt micelle can sustain more cholesterol in the intermicellar aqueous phase than the maximal aqueous solubility. *Arch Biochem Biophys* **20**: 422–477

Ekbom A, Yuen J, Adami H, McLaughlin JK, Chow W, Persson I and Fraumeni JF (1993) Cholecystectomy and colorectal cancer. *Gastroenterology* **105**: 142–147

Garewal H, Bernstein H, Bernstein C, Sampliner R and Payne C (1996) Reduced bile acid-induced apoptosis in 'normal' colorectal mucosa: a potential biological marker for cancer risk. *Cancer Res* **56**: 1480–1483

Giovanni E, Colditz GA and Stampfer MJ (1993) A meta-analysis of cholecystectomy and risk of colorectal cancer. *Gastroenterology* **105**: 130–141

Hirayama T (1989) Association between alcohol consumption and cancer of the sigmoid colon: observations from a Japanese cohort study. *Lancet* **2**: 725–727

Kusche J, Mennigen R, Leisten L and Krahamp B (1988) Large bowel tumor promotion by diamine oxidase inhibition: animal model and clinical aspects. *Agents Actions* **23**: 351–353

Lessof M, Grant V, Hinuma K, Murphy GM and Dowling RH (1990) Recurrent urticaria and reduced diamine oxidase activity. *Clin Exp Allergy* **20**: 373–376

Nagengast FM (1988) Bile acids and colonic carcinogenesis. *Scand J Gastroenterol* **23** (suppl. 54): 76–81

Petersen GM (1995) Genetic epidemiology of colorectal cancer. *Eur J Cancer* **31A**: 1047–1050

Sattler J, Hesterberg R, Chmidt U, Crombach M and Lorenz W (1987) Inhibition of intestinal diamine oxidase activity by detergents: a problem for drug formulations with water insoluble agents applied by the intravenous route? *Agents Actions* **20**: 270–273

Stoneham MD, Young E and Wilkinson JD (1993) Dietary manipulation of fat in chronic urticaria. *J Derm Treat* **4**: 183–185

Wantke F, Demmer CM, Gotz M and Jarisch R (1993) Inhibition of diamine oxidase is a risk in specific immunotherapy. *Allergy* **48**: 552

Winawer SJ, Zauber AG, Gerdes H, O'Brien MJ, Gottlieb LS, Sternberg SS, Bond JH, Waye JD, Schapiro M, Panish JF, Kurtz RC, Shike M, Ackroyd FW, Stewart ET, Skolnick M, Bishop DT and the National Polyp Study Workgroup (1996) Risk of colorectal cancer in the families of patients with adenomatous polyps. *N Engl J Med* **334**: 82–87

© Cancer Research Campaign 1997 


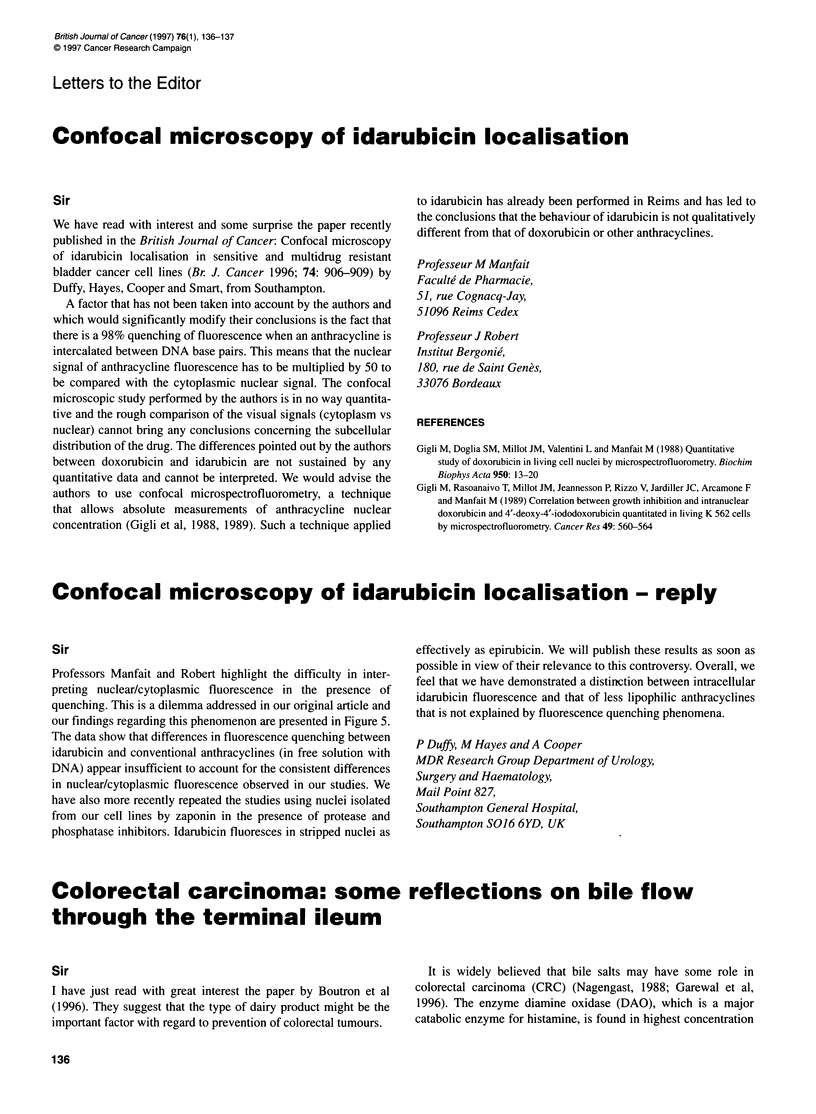

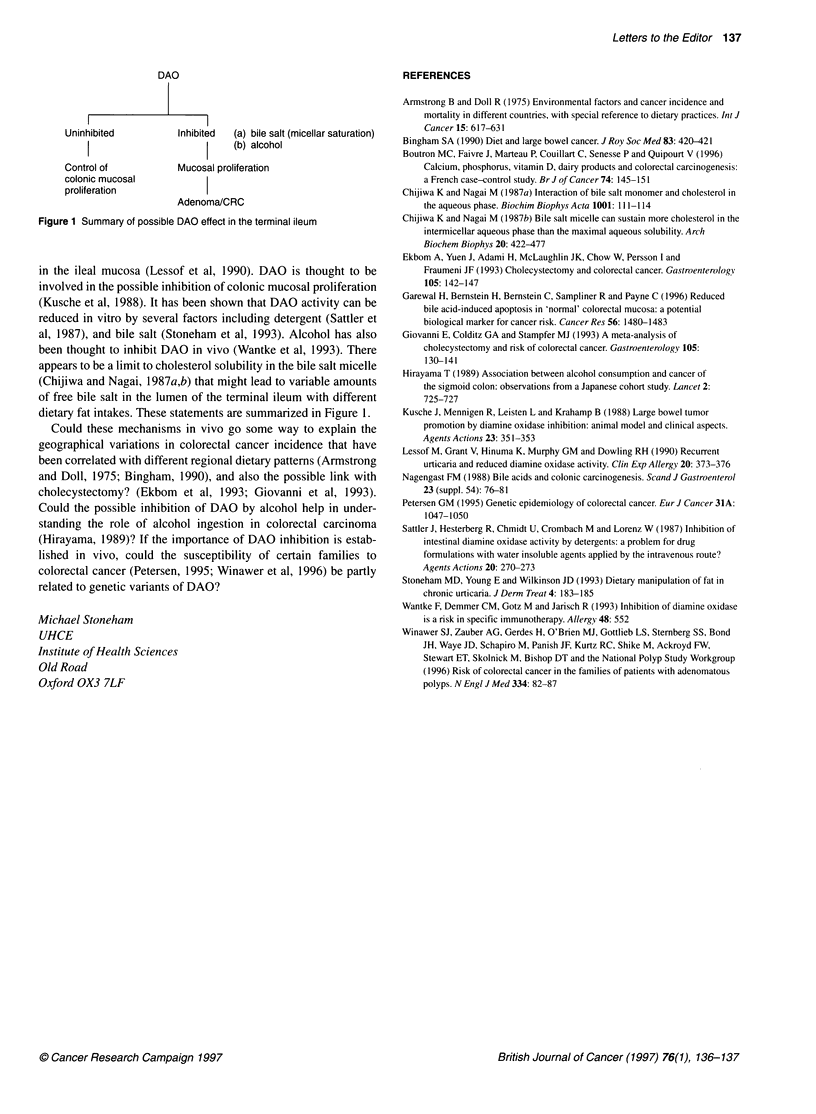


## References

[OCR_00066] Armstrong B., Doll R. (1975). Environmental factors and cancer incidence and mortality in different countries, with special reference to dietary practices.. Int J Cancer.

[OCR_00071] Bingham S. A. (1990). Diet and large bowel cancer.. J R Soc Med.

[OCR_00073] Boutron M. C., Faivre J., Marteau P., Couillault C., Senesse P., Quipourt V. (1996). Calcium, phosphorus, vitamin D, dairy products and colorectal carcinogenesis: a French case--control study.. Br J Cancer.

[OCR_00078] Chijiiwa K., Nagai M. (1989). Interaction of bile salt monomer and cholesterol in the aqueous phase.. Biochim Biophys Acta.

[OCR_00087] Ekbom A., Yuen J., Adami H. O., McLaughlin J. K., Chow W. H., Persson I., Fraumeni J. F. (1993). Cholecystectomy and colorectal cancer.. Gastroenterology.

[OCR_00092] Garewal H., Bernstein H., Bernstein C., Sampliner R., Payne C. (1996). Reduced bile acid-induced apoptosis in "normal" colorectal mucosa: a potential biological marker for cancer risk.. Cancer Res.

[OCR_00097] Giovannucci E., Colditz G. A., Stampfer M. J. (1993). A meta-analysis of cholecystectomy and risk of colorectal cancer.. Gastroenterology.

[OCR_00102] Hirayama T. (1989). Association between alcohol consumption and cancer of the sigmoid colon: observations from a Japanese cohort study.. Lancet.

[OCR_00112] Lessof M. H., Gant V., Hinuma K., Murphy G. M., Dowling R. H. (1990). Recurrent urticaria and reduced diamine oxidase activity.. Clin Exp Allergy.

[OCR_00107] Mennigen R., Kusche J., Krakamp B., Elbers A., Amoei B., Kessebohm M., Sommer H. (1988). Large bowel tumors and diamine oxidase (DAO) activity in patients: a new approach for risk group identification.. Agents Actions.

[OCR_00119] Petersen G. M. (1995). Genetic epidemiology of colorectal cancer.. Eur J Cancer.

[OCR_00123] Sattler J., Hesterberg R., Schmidt U., Crombach M., Lorenz W. (1987). Inhibition of intestinal diamine oxidase by detergents: a problem for drug formulations with water insoluble agents applied by the intravenous route?. Agents Actions.

[OCR_00134] Wantke F., Demmer C. M., Götz M., Jarisch R. (1993). Inhibition of diamine oxidase is a risk in specific immunotherapy.. Allergy.

[OCR_00138] Winawer S. J., Zauber A. G., Gerdes H., O'Brien M. J., Gottlieb L. S., Sternberg S. S., Bond J. H., Waye J. D., Schapiro M., Panish J. F. (1996). Risk of colorectal cancer in the families of patients with adenomatous polyps. National Polyp Study Workgroup.. N Engl J Med.

